# Numerical Simulation of a Scanning Illumination System for Deep Tissue Fluorescence Imaging

**DOI:** 10.3390/jimaging5110083

**Published:** 2019-10-24

**Authors:** Qimei Zhang, Anna M. Grabowska, Philip A. Clarke, Stephen P. Morgan

**Affiliations:** 1 Department of Engineering, Nottingham Trent University, Nottingham NG118PR, UK; qimei.zhang@ntu.ac.uk; 2 School of Medicine, Division of Cancer and Stem Cells, University of Nottingham, Nottingham NG72RD, UK; anna.grabowska@nottingham.ac.uk (A.M.G.); phil.clarke@nottingham.ac.uk (P.A.C.); 3 Optics and Photonics Group, Faculty of Engineering, University of Nottingham, Nottingham NG72RD, UK

**Keywords:** fluorescence imaging, resolution, full-field illumination, near infrared fluorescence and spectral tomography

## Abstract

The spatial resolution and light detected in fluorescence imaging for small animals are limited by light scattering, absorption and autofluorescence. To address this, novel near-infrared fluorescent contrast agents and imaging configurations have been investigated. In this paper, the influence of the light wavelength and imaging configurations (full-field illumination system and scanning system) on fluorescence imaging are compared quantitatively. The surface radiance for both systems is calculated by modifying the simulation tool Near-Infrared Fluorescence and Spectral Tomography. Fluorescent targets are embedded within a scattering medium at different positions. The surface radiance and spatial resolution are obtained for emission wavelengths between 620 nm and 1000 nm. It was found that the spatial resolution of the scanning system is independent of the tissue optical properties, whereas for full-field illumination, the spatial resolution degrades at longer wavelength. The full width at half maximum obtained by the scanning system is 25% lower than that obtained by the full-field illumination system when the targets are located in the middle of the phantom. The results indicate that although imaging at near-infrared wavelength can achieve a higher surface radiance, it may produce worse spatial resolution.

## 1. Introduction

Recent advances in molecular imaging technologies allow detection of physiological and pathological processes in molecular sensitivities from micromolar to picomolar level [1]. Among the molecular imaging modalities, optical imaging methods offer advantages including being relatively inexpensive, rapid and easy to use. Biomedical optical imaging can provide quantitative information on structural, functional, and physiological changes from microscopic to macroscopic levels for objective diagnosis and follow-up [2]. Small animal fluorescence imaging devices can help development and assessment of new drugs, can be used to follow longitudinal changes in diseased tissue and response to intervention and can be used to validate in vitro data and enhance our understanding of in vivo events. As a noninvasive method it eliminates the need to obtain post-mortem tissue from animals therefore reduce the number of animals used. Nevertheless, in vivo fluorescence imaging is expected to have a significant impact on animal based research [3].

The spatial resolution and light detected by the in vivo fluorescence imaging are however limited by light scattering, absorption and autofluorescence in tissue. Scattering acts on both the excitation and emission light and introduces an intrinsic blurring in fluorescence images [4]. Another effect of scattering is it results in reduced penetration depth, which is also limited by absorption of light by tissue components for macroscopy imaging [5]. Autofluorescence interferes with and obscures the detection of extrinsic fluorescent contrast agents, manifesting as a high background signal in an image that reduces contrast and clarity.

Development of near-infrared (NIR) contrast agents for fluorescence imaging has attracted much interest [6,7,8,9], because the 650–1000 nm wavelength range has lower scattering and absorption coefficient [10]. In addition, the tissue autofluorescence spectrum is mostly in the visible light wavelength range and reduces significantly in the NIR wavelength range above 700 nm [4]. Light in the NIR wavelength range has higher penetration depth. A large amount of research has focused on investigating novel NIR fluorescent contrast agents to improve sensitivity and obtain accurate quantitative images in vivo [4,6,7,8,9,10].

Planar imaging is a simple, inexpensive and rapid way to image the fluorescence. Generally planar fluorescence imaging configurations for small animals can be divided into two categories based on source arrangements. The most widely used system is full-field illumination imaging, in which an excitation light source (e.g., light-emitting diode (LED), lamp or laser) supplies a full field excitation and the images are captured with a camera (e.g., the IVIS series from PerkinElmer). An alternative imaging configuration is a scanning laser system in which a galvonometer-based scanner scans across and illuminates the sample, and the emission light is captured by a photomultiplier tube (PMT), an avalanche photodiode (APD) or a charge-coupled device (CCD) camera [4]. In general, the measurements are acquired in either epi-illumination mode (i.e., reflectance) or trans-illumination mode (i.e., transmission).

Spatial frequency domain imaging (SFDI) is a full-field illumination method that can enhance the spatial resolution of fluorescence imaging [11]. In SFDI, a spatially modulated light source is projected into tissue as the excitation light. The tissue acts as a low pass filter that leads to a smaller penetration depth for a higher modulation frequency [12]. By using differing spatial frequencies, the fluorescence signal from deeper regions can be diminished and the signal close to the surface can be enhanced [11]. However, SFDI is only sensitive to a depth of a few milimeters [11].

There have also been several techniques based on scanning illumination beam proposed to enhance resolution and depth of fluorescence imaging. In Laminar Optical Tomography [13], a focused beam of light scans across the surface of the tissue by using galvanometer mirrors. A linear array of detectors is used to detect light from both the focus of the scanning beam and also at increasing distances from the beam’s focus. Light detected further away from the focus travelled deeper inside tissue therefore can provide depth-resolved information. In the configuration described in [14,15], a laser line scans across the sample and the fluorescence signal is detected by a CCD camera producing a stack of images. One single stripe is obtained from each of the image and stripes are concatenated to form the whole field of view. The selection of photons reduced the negative effects of background signal and scattering. Images with higher contrast and resolution within a few millimetres of tissue are therefore obtained.

Although contrast and resolution of images are improved in [14,15], there are still aspects to investigate about the scanning approach. The laser line scanning is a faster scanning method compared with the point scanning approach. However, the configuration described in [14,15] requires post-processing to concatenate the stripes obtained by the CCD for each scanning position. A point scanning approach is much simpler in terms of imaging processing method. In addition, the optical properties of tissue are wavelength dependent and it is interesting to investigate how wavelength can affect imaging quality for the scanning approach. Although the advantages and disadvantages of scanning (e.g., laser scanning confocal microscopy) and full-field microscope systems are well established this has not been widely investigated in heavily scattering media where light cannot be focused. A model of the imaging technique in multiply scattering media can help to understand the effects of imaging configurations over a wide range of wavelengths. The model also allows us to investigate different configurations which can help inform future device design.

In this paper, a fast modelling technique based on the finite element method (FEM) for a point scanning fluorescence imaging system is developed. The influence of the two aspects, light wavelength and imaging configuration, on the fluorescence imaging surface radiance and spatial resolution are studied quantitatively. Surface radiance for the scanning imaging system is calculated and compared with a full-field illumination system. The ability to resolve two fluorescent targets using these two imaging systems in both reflection and transmission mode are compared.

## 2. Methods

### 2.1. Simulation Methods

**Figure 1:**
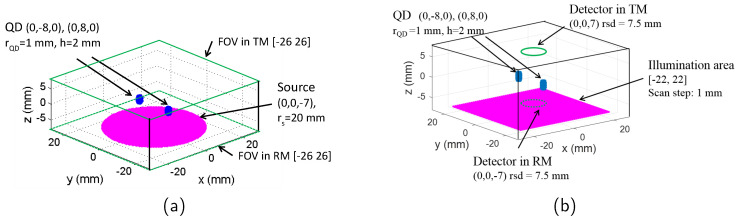
Simulation geometry of (**a**) full-field illumination system and (**b**) scanning system. FOV: field of view; TM: transmission mode; RM: reflection mode; QD: target containing quantum dots.

To obtain the imaging resolution and surface radiance of the full-field and scanning mode imaging, it is necessary to obtain the photon fluence distribution within the simulated tissue first. This is achieved numerically by modifying the tool Near Infrared Fluorescence and Spectral Tomography (NIRFAST) [16]. As an open source software based on FEM, NIRFAST can model multiwavelength and luminescence (fluorescence and bioluminescence) light propagation through tissues of arbitrary shapes in continuous wave, time domain or frequency domain [16,17]. The forward model of NIRFAST simulates diffusely propagating light during excitation and emission by calculating the diffusion approximation of the radiative transport equation [16]. The simulation geometries used are shown in Figure 1. The tissue-like phantom has a size of 55–55–15 mm (x–y–z); 15 mm is selected as it is a typical depth (ventral to dorsal) for an adult laboratory mouse which has a mean weight around 30 g. NIR emitting fluorophores such as lead-based quantum dots (QDs) have gained considerable attention in the last decade, because their emission wavelength can be tuned from 750 nm to 3700 nm which makes them viable for bioimaging application [18]. The fluorescent targets used in the simulations in this paper are therefore modelled as a cluster of QDs which would be observed if they become localised in a region of tissue such as in a tumour or an organ. The QDs regions are simulated as two cylinders with the same size (radius $r_{QDs}=1\;\mathrm{mm}$, height h=2 mm). This size is small enough to investigate the imaging spatial resolution while it also satisfies the Nyquist theorem, by being more than 2 times larger than the phantom FEM mesh size of 0.3 mm. The geometry centres of the two targets are located at (0, −8, 0) and (0, 8, 0) (unit: mm) inside the phantom, respectively.

#### 2.1.1. Full-Field Illumination System

The source for the full-field illumination system is a circular shape (typically used in systems such as the IVIS Spectrum by Perkin Elmer) with the centre located at (0, 0, −7) mm and radius $r_{s}=20\;\mathrm{mm}$, as seen in Figure 1. The meshes of phantom, QDs region, and the illumination source are generated using the *creat mesh* function of NIRFAST. The surface radiances (unit: $\mathrm{photons}\;s^{-1}\;{\mathrm{mm}}^{2}\;\mathrm{sr}$) at the top surface (for transmission mode, in the area of [−26, 26], z=7 mm) and bottom surface (for reflection mode, in the area of [−26, 26], z=−7 mm) are calculated based on the forward model of NIRFAST.

Specifically, the surface radiance is calculated in three steps: (a) calculate the photon fluence all over the regions where the QDs are located using the forward model of NIRFAST, (b) convert the photon fluence at the QDs regions to their emission strength and (c) calculate the surface radiance at the detector for both the reflection mode and transmission mode with the previously calculated fluorescence emission as the new source for the forward model of the NIRFAST. The emission strength ($S^{fl}$) at step b is calculated by the following equation [19],

(1) $$S^{fl}\left(r\right)=\Phi \xi^{ex}N_{0}A^{ex}\left(r\right)$$

where Φ is the quantum yield of the QDs (in fraction), $\xi^{ex}$ is the extinction coefficient of the QDs at the excitation wavelength ( m2), $N_{0}$ is the quantum dots concentration ( m-3) and $A^{ex}$ is the photon fluence of the excitation light at the region where the QDs are located calculated at step a.

#### 2.1.2. Scanning System

The simulation geometry of the scanning system is shown in Figure 1b. Light from a point source illuminates normally the tissue slab and it is scanned in x–y plane (z = −7 mm) across an area of [−22, 22] mm with a step of 1 mm. The power density of the illumination is assumed to be identical to the full-illumination system. The detector is a circular shape with a radius of $r_{sd}=7.5\;\mathrm{mm}$, and centre located at (0, 0, 7) mm for transmission mode and (0, 0, −7) mm for reflection mode. For each scanning position of the source, the surface radiance over the detecting area is integrated.

The most time-consuming aspect of the simulation is to calculate the fluence once the source, detector and phantom properties have been decided. For example, the time taken to calculate the fluence at the targets with the computing facility of one core processor (Intel Sandybridge E5-2670, 2.6 GHz) and 24 GB memory is ~ 35 min for the geometry described in Figure 1b and one scan position. Therefore, it would take at least 98 days to obtain one scanning image through the three steps method mentioned earlier. To increase the simulation speed of the scanning imaging, an equivalent calculation method is used. The fluence of the light source can be calculated only once for each simulated optical wavelength. For the simulated scanning system, it can be assumed that scanning the excitation beam is equivalent to scanning the QDs region and the detector together as it is only the relative positions of source, object and detector that affects the final image. This is because the phantom simulated has homogeneous optical properties outside the target region and the size of the phantom ([−27.5, 27.5] mm) is bigger than the illuminated region ([−22, 22] mm), and therefore can be regarded as an infinite slab. Since the relative position of the QDs region and the detector does not change, the integrated intensity of the detector is always proportional to the emission strength of the QDs. Based on Equation (1), the integrated intensity is also proportional to the fluence of the excitation light at the QDs region. A reflection factor (transmission factor) is therefore defined as the ratio of the light intensity at the detector in reflection mode (transmission mode) with the photon fluence of the excitation light arrived at the QDs region. The reflection and transmission factor are constants for the same emission wavelength. In the simulation, the light intensity for transmission and reflection mode are calculated respectively only once with the point source located at (0, 0, −7) mm. The transmission factor, reflection factor and photon fluence distribution in the phantom can all be obtained from this calculation. For each scanning position of the excitation beam in the illumination area, the excitation light arrived at the QDs can be obtained by firstly acquiring the corresponding photon fluence based on its spatial location relative to the excitation beam, and then converting the photon fluence to the light intensity by multiplying either the reflection factor (for reflection mode) or transmission factor (for transmission mode). The simulation time for one scan image with this method is ~6 h, therefore the simulation speed is reduced by a factor of approximately 400 times. Typical parameter values of the QDs are used in the simulation and are summarised in Table 1.

### 2.2. Calculation of Optical Properties

The reduced scattering coefficient ($\mu_{s}^{{}^{\prime }}$) of a tissue can be obtained using [22]

(2) $$\mu_{s}^{\prime }\left(\lambda \right)=a{\left(\frac{\lambda }{500\;\left(\mathrm{nm}\right)}\right)}^{-b}$$

where λ is the wavelength, $a=\mu_{s500\;\left(\mathrm{nm}\right)}^{{}^{\prime }}$ is the reduced scattering coefficient at 500 nm and *b* is the scattering power.

The absorption coefficient of a tissue is the sum of contributions from all absorbing chromophores within the tissue and can be expressed as [22]

(3) $$\begin{array}{cc} \mu_{a}\left(\lambda \right) & =BS\mu_{a,oxy}+B\left(1-S\right)\mu_{a,deoxy}+WS\mu_{a,water}\\ \; & +FS\mu_{a,fat}+MS\mu_{a,mel} \end{array}$$

where *S* is the oxygen saturation of hemoglobin, *B*, *W*, *F*, *M* are the volume fraction of blood, water, fat and melanosome, respectively. $\mu_{a,oxy}$, $\mu_{a,deoxy}$, $\mu_{a,water}$, $\mu_{a,fat}$, and $\mu_{a,mel}$ are the absorption coefficients of oxygenated blood, deoxygenated blood, water, fat and melanosome, respectively.

**Figure 2:**
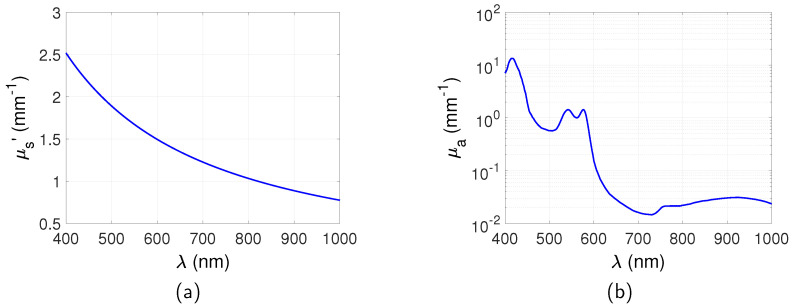
Spectra of (**a**) reduced scattering coefficient and (**b**) absorption coefficient, obtained based on Equations (2) and (3). $a=1.89mm^{-1}$, b=1.286, B=0.0511, S=0.8 [22]. $\mu_{a,oxy}$ and $\mu_{a,deoxy}$ are obtained from the authors of [23].

It can be seen from Equations (2) and (3) that $\mu_{s}^{{}^{\prime }}$ and $\mu_{a}$ are a function of the wavelength. Simulation of the excitation and emission wavelength of the targets is therefore through a corresponding calculation of the reduced scattering coefficient and absorption coefficient. This is an ideal wavelength filtering situation that only the emitted light is detected from the QDs. $\mu_{s}^{{}^{\prime }}$ and $\mu_{a}$ used in these simulations were obtained based on Equations (2) and (3) and are shown in Figure 2. $a=1.89\;{\mathrm{mm}}^{-1}$, b=1.286, B=0.0511, S=0.8 [22]. $\mu_{a,oxy}$ and $\mu_{a,deoxy}$ are obtained from the authors of [23]. Only blood component is considered for $\mu_{a}$ between 400 nm and 1000 nm as this is the dominant absorber in this wavelength region. Water may become dominant over blood at wavelengths longer than 1000 nm but is much lower between 400 nm and 1000 nm [22] and is neglected in these simulations. Similarly the absorption of fat and melanosome is low in this range and is neglected [22].

### 2.3. Spatial Resolution

The spatial resolution is defined as the minimum separation of two targets that can still be perceived as separate by an observer. The spatial resolution is estimated by the Sparrow criterion [24]: the minimum separation is achieved when the position at which two point spread functions overlap equals the full width at half maximum (FWHM). In the simulation, the FWHM for each of the targets is calculated through the calculation of the surface radiance distribution using NIRFAST and then the analysis of the signal profile along one dimension. The averaged FWHM of the two targets is regarded as the minimum separation as the targets are identical. This is valid for the case of the Born approximation for the heterogeneous diffusion equation in which the detected light is obtained from a linear superposition of the light propagating from source to target and then onto the detector [25,26].

### 2.4. Simulation of Autofluorescence

Within the 200–650 nm excitation wavelength range, the autofluorescence from tissue also confounds fluorescence measurements. In this paper, the autofluorescence is simulated by treating the intrinsic fluorophores that are responsible for generating autofluorescence similarly to QDs. The intrinsic fluorophores have an intrinsic fluorescence coefficient ($\beta (\lambda_{ex},\lambda_{em})$), which is defined as [27]

(4) $$\beta \left(\lambda_{ex},\lambda_{em}\right)\equiv \mu_{a,AF}\left(\lambda_{ex}\right)\Phi_{\lambda }\left(\lambda_{em}\right)$$

where $\mu_{a,AF}\left(\lambda_{ex}\right)$ is the absorption coefficient of the intrinsic fluorophores at the excitation wavelength. $\Phi_{\lambda }\left(\lambda_{em}\right)$ is the dimensionless spectral fluorescence energy yield of the intrinsic fluorophores at the emission wavelength. Note that $\mu_{a,AF}\left(\lambda_{ex}\right)$ is only a fraction of the total absorption coefficient of the medium which is much larger.

The autofluorescence emission strength can thus be calculated as

(5) $$S^{AF}\left(r\right)=\beta A^{ex}\left(r\right)$$

In the simulation the intrinsic fluorophores are simulated as the nodes of a mesh which has the same size as the tissue slab but with a bigger node distance of 5 mm to reduce the storage memory and increase the calculation time. The intrinsic fluorescence coefficient at the excitation wavelength of $\lambda_{ex}=600\;\mathrm{nm}$ was obtained from the literature [27].

## 3. Results

### 3.1. Maps of Photons Distribution

**Figure 3:**
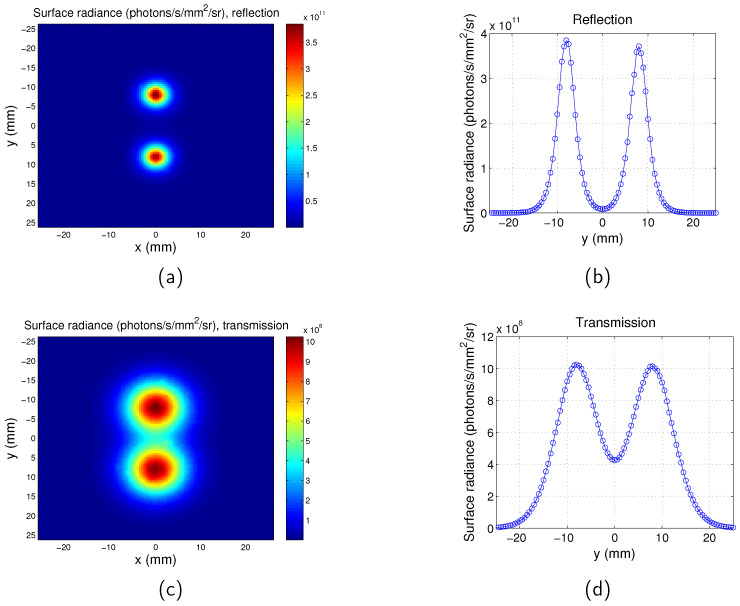
Surface radiance for full-field illumination imaging system. z=−4 mm. Separation between the two targets is D=16 mm. $\lambda_{ex}=600\;\mathrm{nm}$. $\lambda_{em}=620\;\mathrm{nm}$. (**a**) Map for reflection mode; (**b**) surface radiance along *y* direction at x=0 mm for reflection mode; (**c**) map for transmission mode; and (**d**) surface radiance along *y* direction at x=0 mm for transmission mode.

The surface radiance at the plane of the detector for the full-field illumination system is calculated for both the transmission mode and reflection mode. An example is shown in Figure 3, with the targets located at the plane z=−4 mm and the separation between the two targets D=16 mm. $\mu_{s}^{{}^{\prime }}=1.49\;{\mathrm{mm}}^{-1}$ and $\mu_{a}=0.1504\;{\mathrm{mm}}^{-1}$ at the excitation wavelength $\lambda_{ex}=600\;\mathrm{nm}$. $\mu_{s}^{{}^{\prime }}=1.43\;{\mathrm{mm}}^{-1}$ and $\mu_{a}=0.0563\;{\mathrm{mm}}^{-1}$ at the emission wavelength $\lambda_{em}=620\;\mathrm{nm}$. As mentioned in Section 2.1, the thickness of the phantom is 15 mm, therefore the depth of the targets for this case is 3.5 mm beneath the excitation source plane, and the distance between the targets and the opposite plane is 11.5 mm. It is clear that the targets are closer to the source plane rather than the opposite plane, therefore the two targets can be better distinguished by reflection mode imaging.

**Figure 4:**
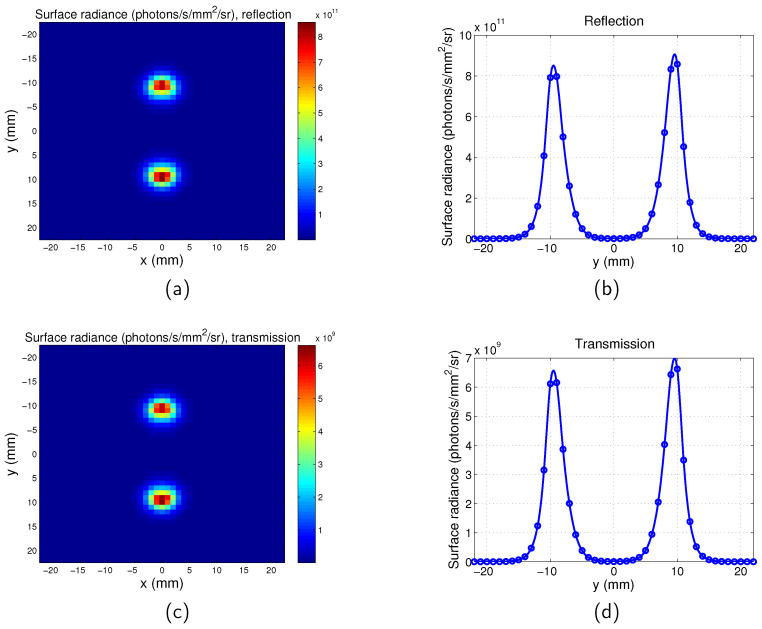
Surface radiance for scanning imaging system. z=−4 mm. Separation between the two targets is D=16 mm. $\lambda_{ex}=600\;\mathrm{nm}$. $\lambda_{em}=620\;\mathrm{nm}$. (**a**) Map for reflection mode; (**b**) surface radiance along *y* direction for x=0 mm for reflection mode; (**c**) map for transmission mode; and (**d**) surface radiance along *y* direction for x=0 mm for transmission mode.

Similarly, the surface radiance distribution for the scanning system of both the transmission mode and reflection mode are shown in Figure 4 with z=−4 mm and distance between the two targets D=16 mm. It can be seen that the spatial resolutions of the reflection and transmission systems are very similar. Comparison of Figure 4 with Figure 3 suggests that the two targets can be better distinguished by the scanning imaging system. The pixel size of the scanning system is 1 mm and it is determined by the scan step. The maps of the full-field illumination system are plotted based on the nodes of the mesh, therefore the pixel size of the full-field illumination system is determined by the mesh size of the phantom ( 0.3 mm). This leads to the images obtained from the scanning system being more pixelated than that of the full-field illumination system. The tetrahedral mesh of the phantom means that the two targets are not exactly symmetrical to the y = 0 mm plane, therefore there is a slight difference between the values of the two peaks in one image.

### 3.2. Surface Radiance

**Figure 5:**
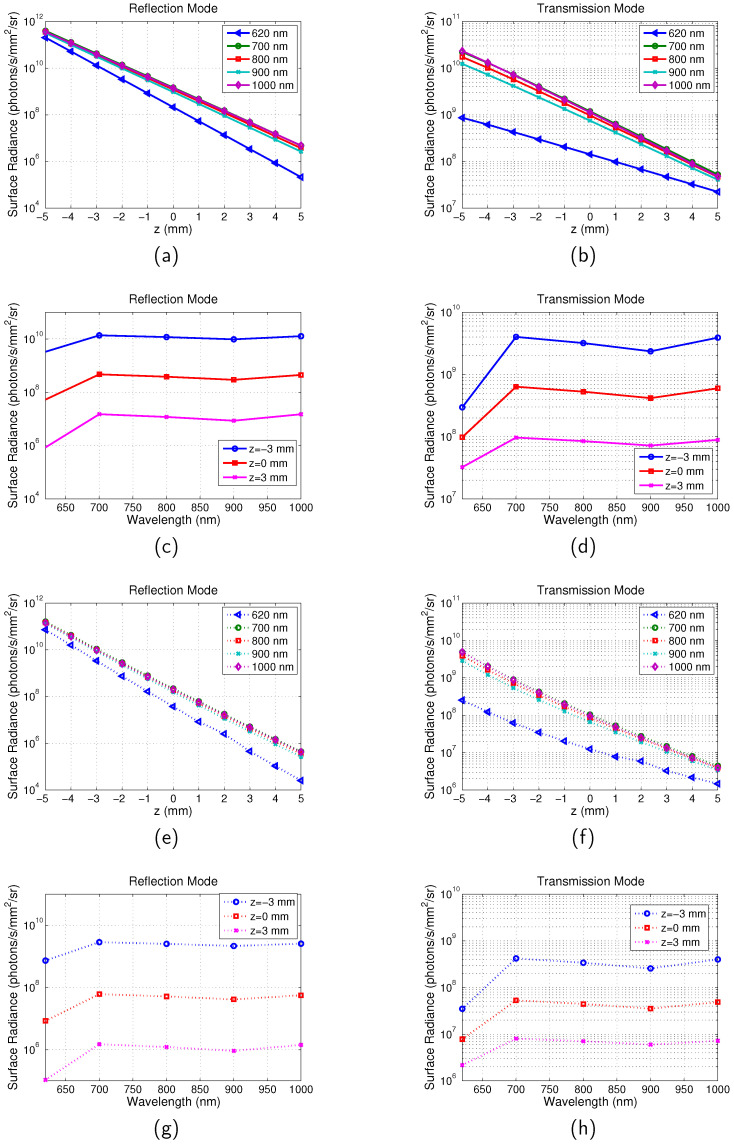
Calculation of Surface radiance. Optical properties are the same as those indicated by Figure 2. Separation between the two targets is D=16 mm. $\lambda_{ex}=600\;\mathrm{nm}$. (**a**) Surface radiance vs. *z* for reflection mode of full-field system. (**b**) Surface radiance vs. *z* for transmission mode of full-field system. (**c**) Surface radiance vs. $\lambda_{em}$ for reflection mode of full-field system. (**d**) Surface radiance vs. $\lambda_{em}$ for transmission mode of full-field system. (**e**) Surface radiance vs. *z* for reflection mode of scanning system; (**f**) Surface radiance vs. *z* for transmission mode of scanning system. (**g**) Surface radiance vs. $\lambda_{em}$ for reflection mode of scanning system. (**h**) Surface radiance vs. $\lambda_{em}$ for transmission mode of scanning system.

The averaged surface radiance of all the pixels across the surface for full-field illumination system and scanning system is shown in Figure 5. The surface radiance is calculated as a function of the depth of the target *z* in the range between −5 mm and 5 mm with a step of 1 mm and emission wavelength in the range between 620 nm and 1000 nm. From Figure 5a,b, it can be seen that with increase of *z*, the surface radiance for both the reflection and transmission mode decreases. This is due to the increased distance between the illumination source and the targets. Longer distance means that more excitation photons are absorbed or scattered along the path and therefore less excitation light reaches the targets. It is also found that the depth change has greater influence on reflection than transmission. For example, for the imaging with the scanning system and at the emission wavelength of 700 nm, the surface radiance decreased $3.3\times 10^{5}$ times for the reflection mode and $1.3\times 10^{3}$ times for the transmission mode. This can be understood that the photon density of the excitation light is higher near the incident boundary. Similar results were observed in [28]. The dependence of the surface radiance on wavelength is shown in Figure 5c,d. It can be seen that it is in accordance with the change of $\mu_{a}$ with the emission wavelength ($\mu_{a}=0.0545\;{\mathrm{mm}}^{-1}$ at $\lambda_{em}=620\;\mathrm{nm}$; $\mu_{a}=0.0162\;{\mathrm{mm}}^{-1}$ at $\lambda_{em}=700\;\mathrm{nm}$; $\mu_{a}=0.0220\;{\mathrm{mm}}^{-1}$ at $\lambda_{em}=800\;\mathrm{nm}$; $\mu_{a}=0.0304\;{\mathrm{mm}}^{-1}$ at $\lambda_{em}=900\;\mathrm{nm}$; $\mu_{a}=0.0236\;{\mathrm{mm}}^{-1}$ at $\lambda_{em}=1000\;\mathrm{nm}$). From $\lambda_{em}=700\;\mathrm{nm}$ to 900 nm $\mu_{a}$ increases leading to a decreased surface radiance. From $\lambda_{em}=900\;\mathrm{nm}$ to 1000 nm, $\mu_{a}$ decreases leading to an increased surface radiance. However these are much smaller effects compared to the effect of *z*.

### 3.3. Spatial Resolution

**Figure 6:**
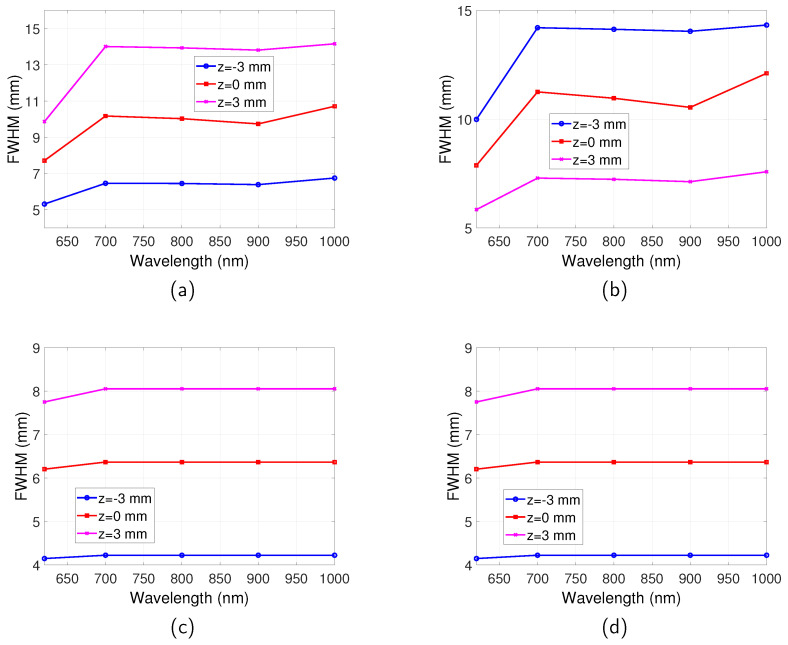
Spatial resolution for full-field illumination system and scanning system as a function of wavelength. Optical properties are the same as those indicated by Figure 2. Separation between the two targets is D=16 mm. $\lambda_{ex}=600\;\mathrm{nm}$. (**a**) FWHM vs. $\lambda_{em}$ for full-illumination system in reflection mode. (**b**) FWHM vs. $\lambda_{em}$ for full-illumination system in transmission mode. (**c**) FWHM vs. $\lambda_{em}$ for scanning system in reflection mode. (**d**) FWHM vs. $\lambda_{em}$ for scanning system in transmission mode.

The spatial resolutions for the full-field illumination system and scanning system, in both reflection and transmission mode, are shown in Figure 6. For the full-field illumination system, the dependence of the spatial resolution on wavelength is found to be in accordance with the relationship of $\mu_{a}$ with the emission wavelength. The best spatial resolution (smallest FWHM) is obtained at $\lambda_{em}=620\;\mathrm{nm}$ where $\mu_{a}$ is the highest. The FWHM decreases in the range between $\lambda_{em}=700\;\mathrm{nm}$ and 900 nm where $\mu_{a}$ increases. The FWHM increases from $\lambda_{em}=900\;\mathrm{nm}$ to 1000 nm where $\mu_{a}$ decreases. Therefore, it can be summarised that the spatial resolution is better for a higher $\mu_{a}$. This can be understood that $\mu_{a}$ stops photons with long trajectories that degrade spatial resolution reaching the boundary.

For the scanning system, an observation from Figure 6 suggests that the transmission and reflection mode imaging have the same spatial resolution, which are both much better than that of the full-field illumination system. The emission wavelength also has very little influence on the spatial resolution for all the wavelengths that were studied.

**Figure 7:**
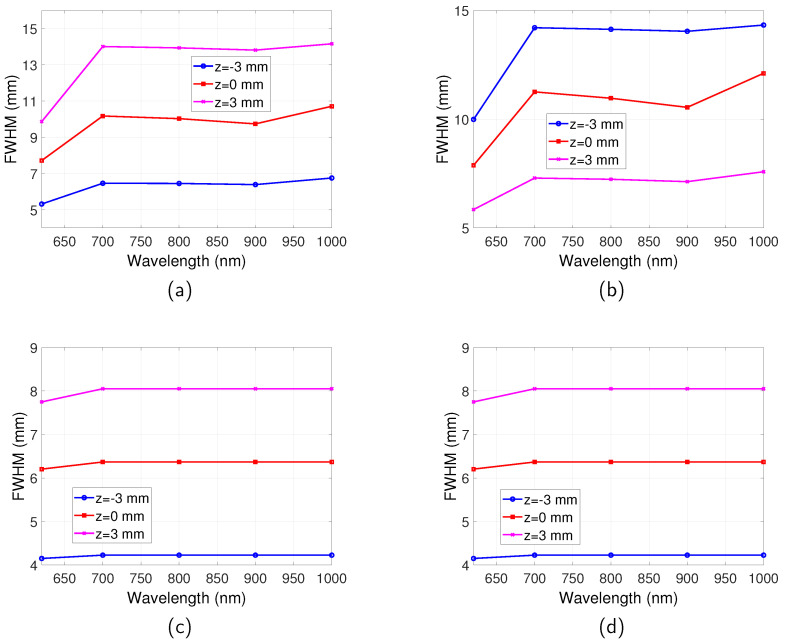
Spatial resolution for full-field illumination and scanning system as a function of *z* position. (**a**) FR: full-field illumination system reflection mode. (**b**) FT: full-field illumination system transmission mode. (**c**) S: scanning system. Optical properties are the same as those indicated by Figure 2. Separation between the two targets is D=16 mm. $\lambda_{ex}=600\;\mathrm{nm}$.

The dependence of spatial resolution on depth of fluorescent targets can be more clearly observed from Figure 7, where the FWHM of full-field illumination system reflection/transmission mode imaging and scanning system imaging are plotted as a function of *z*. For the full-field illumination system detecting in reflection mode (Figure 7a), it can be seen that with increase of *z*, where the targets are further away from both the illumination source and the detector, the FWHM increases. On the other hand, the spatial resolution in transmission mode improves, as suggested by Figure 7b. For the case when the targets are situated in the middle of the tissue slab, the same spatial resolution is obtained for reflection and transmission mode and it is the worst spatial resolution for all the buried depths.

For the scanning system as shown in Figure 7c, it is found that with increase of *z* the spatial resolution gets worse. The closer the targets are to the excitation light, the better the spatial resolution. It can also be observed by comparing Figure 7c with Figure 7a,b that generally the scanning system has a better spatial resolution than the full-field illumination system.

**Figure A1:**
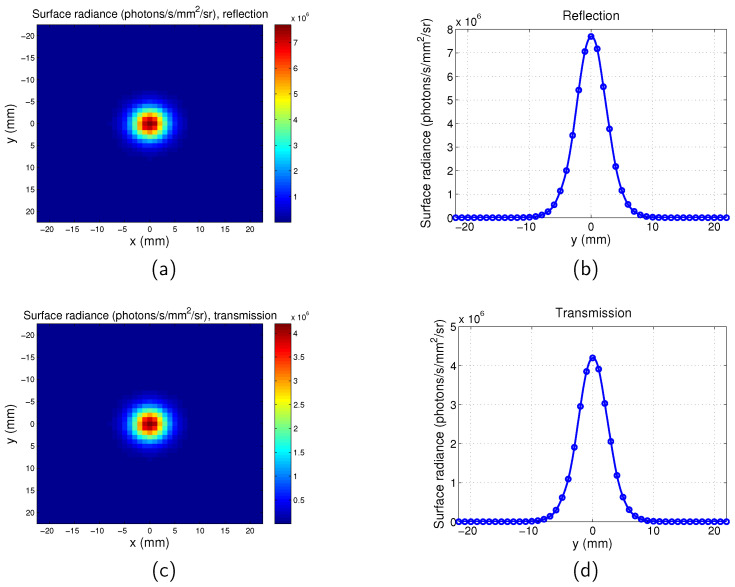
Point spread function for scanning imaging system. z=−1 mm, $\lambda_{ex}=600\;\mathrm{nm}$, $\lambda_{em}=1000\;\mathrm{nm}$. (**a**) Map for reflection mode; (**b**) surface radiance along *y* direction for x=0 mm for reflection mode; (**c**) map for transmission mode; and (**d**) surface radiance along *y* direction for x=0 mm for transmission mode.

**Figure A2:**
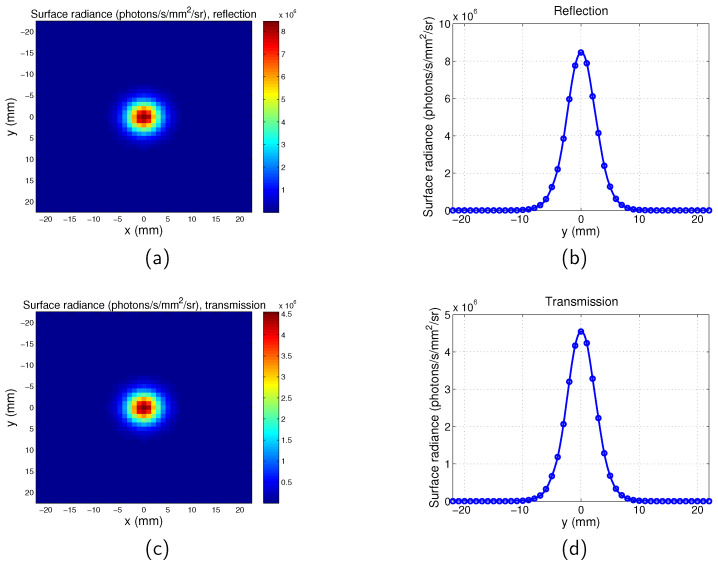
Point spread function for scanning imaging system. z=−1 mm, $\lambda_{ex}=600\;\mathrm{nm}$, $\lambda_{em}=700\;\mathrm{nm}$. (**a**) Map for reflection mode; (**b**) surface radiance along *y* direction for x=0 mm for reflection mode; (**c**) map for transmission mode; and (**d**) surface radiance along *y* direction for x=0 mm for transmission mode.

Although the emission wavelength has no influence on the spatial resolution of the scanning system, the illumination light does affect the spatial resolution. To demonstrate this, a range of excitation wavelengths ( 600 nm, 700 nm, 800 nm and 900 nm) is simulated with a fixed emission wavelength of 1000 nm. Point spread function (PSF) is calculated for each of the excitation wavelength and FWHM of the PSF is used to estimate the resolution. The PSF here is defined as the detected image when the quantum dot is a point target. Example PSFs for the scanning system is shown in Figure A1 and Figure A2 in Appendix A. The estimated resolutions for the simulated wavelengths are summarised in Table A1 in Appendix A. It can be seen that the resolution is mainly determined by the absorption coefficient determined by the excitation light—the resolution is better for a higher absorption coefficient.

### 3.4. Influence of Autofluorescence on Surface Radiance and Spatial Resolution

**Figure 8:**
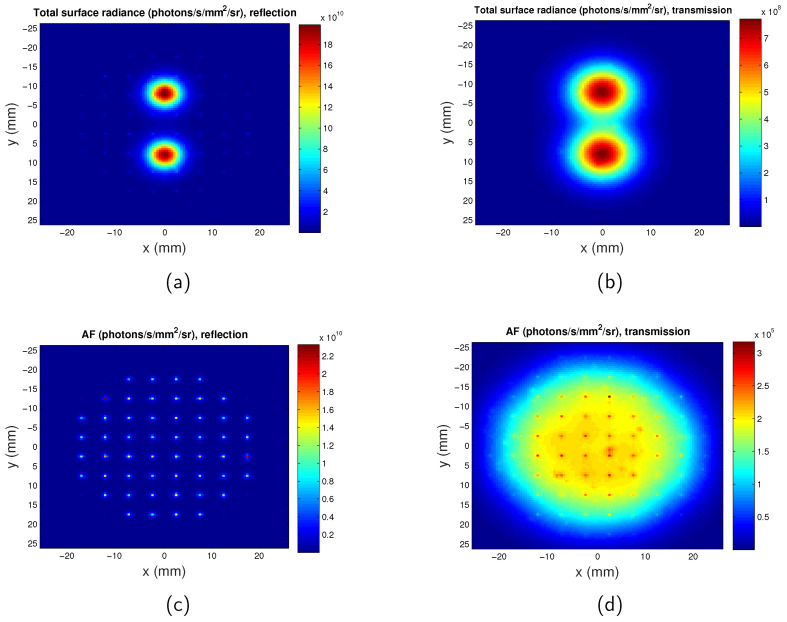
Maps of surface radiance considering autofluorescence at excitation wavelength of $\lambda_{ex}=600\;\mathrm{nm}$. z = −4 mm. Separation between the two QDs regions is D=16 mm. (**a**) Total surface radiance in reflection mode; (**b**) total surface radiance in transmission mode; (**c**) surface radiance for only autofluorescence in reflection mode; and (**d**) surface radiance for only autofluorescence in transmission mode. AF: autofluorescence.

The surface radiance of the full-field illumination system is calculated when autofluorescence is considered, as shown in Figure 8. It can be seen that at the excitation wavelength of 600 nm, the autofluorescence light intensity detected is 8.5 times dimmer than that of the targets. As would be anticipated at such long wavelengths, autofluorescence does not have a significant effect on the surface radiance. Autofluorescence increases background light but does not influence spatial resolution therefore its effect is also not considered.

For the scanning system, when the scanning source is located directly opposite to the detector, the photon intensity due to autofluorescence detected is at its maximum. The maximum intensity due to autofluorescence in transmission mode and reflection mode are calculated to be $2.3\times 10^{6}$ and $9\times 10^{4}$ times dimmer than the intensity generated by the targets, respectively. Therefore, for the scanning system, the influence of the autofluorescence can also be ignored.

## 4. Discussions

Much attention has been focused on developing novel probes emitting light in the NIR spectral window, because tissue has minimal absorbance in the wavelength range between 650 nm and 1000 nm, allowing for deep penetration depth and higher sensitivity [7]. The findings in this paper suggest that imaging at near-infrared wavelength can indeed obtain higher levels of surface radiance compared with visible light at the same depth, and near-infrared light has better penetration depth. However, fluorophores at the near-infrared window can degrade spatial resolution mainly due to lower absorption. This is because the lower absorption means that photons that have propagated on longer paths are not heavily attenuated. For example, for transmission mode imaging of full-field illumination system at z = −5 mm, the FWHM is 15.0 mm instead of 11.7 mm if using fluorescent probes with 1000 nm emission wavelength rather than 620 nm emission wavelength. Therefore, there is a compromise between the penetration depth and the spatial resolution. Only absorption due to blood components which are the dominant factors is considered in the simulation. The effect of the other absorbers such as water, fat and melanosome is to increase slightly the absorption coefficient depending on the specific tissue. Different tissues have different absorbing chromophores and concentration therefore have different $\mu_{a}$ [22]. However, a range of $\mu_{s}^{\prime }$ and $\mu_{a}$ are simulated, and the effect of the other absorbers is only a shift of the simulated $\mu_{a}$ to other values, which will not affect the overall conclusions of this work.

Improvement of spatial resolution can be obtained by using a scanning system, which has a much better spatial resolution than the full-field illumination system. For example, for the case when the targets are located in the middle of the phantom, the FWHM obtained by the full-field illumination system is 8 mm, whereas that obtained by the scanning system is 6 mm which is 25% lower. The spatial probability distribution of photons entering tissue at a source location, scattering through the tissue, and being emitted at a particular detector location, defines the spatial sensitivity profile for that source-detector pair [29]. A typical sensitivity profile can be found in Ref. [30]. The sensitivity profile can be used to indicate spatial resolution—targets located at narrower position of the profile can be better distinguished. The excitation light of the full-field illumination system has a large field ( 20 mm radius), therefore the closer the targets are to the detector, the narrower the profile and the better the spatial resolution, as shown in Figure 7a,b. For the scanning system, because a point excitation source is used, the sensitivity profile is narrower and the spatial resolution is better. Such capabilities can now be found in current imaging platforms, such as the IVIS Spectrum (Perkin Elmer), but these at present do not include spatial filtering at the detector plane. A similar conclusion that a scanning system provides better spatial resolution than wide field illumination system is found by Fantoni [14]. However, in this study, a line scanning method is used and the wavelength range considered is much smaller.

It was found that for the scanning system, the same spatial resolution is obtained for reflection mode and transmission mode. The emission wavelength of the fluorescent probe also has no effect on the spatial resolution. The spatial resolution is only dependent on the distance between the excitation source and the targets. This is because the $\mu_{a}$ of emission works as a scaling factor and only affects the photon fluence detected; the FWHM remains the same. The increase of $\mu_{s}^{\prime }$ changes the exit position of the emitted photons, but due to an integrated detector ( 7.5 mm in radius) applied in this case, the spatial resolution stays the same. The spatial resolution depends on the spatial light distribution at the object plane which depends on the optical parameters of excitation rather than emission.

**Figure A3:**
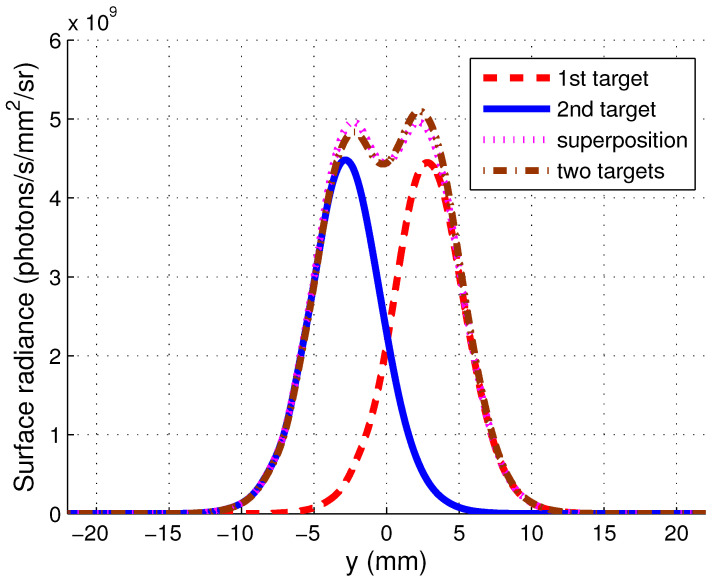
Spatial distribution of fluorescence emission light from fluorescent targets.

In the simulation, the resolution is estimated as the average value of the FWHMs of the two fluorescent targets with an arbitrary separation of 16 mm. A change of the separation will not influence on the resolution. To demonstrate that this method of estimating resolution is accurate within the Born approximation, the light distribution of the two fluorescent targets are calculated separately and then superimposed, followed by an image of two targets. The fluorescence light distribution of the first target, the second target, the superposition of the two images of the single targets and the fluorescence light distribution of the two targets are shown in Figure A3 in Appendix A. It can be seen that the cases of the superposition and the two targets are very close to each other. The resolution obtained for the linear superposition case is 5.73 mm. Therefore, the coordinates of the targets in the combined image are chosen as [0,−2.865 mm,−1] and [0,2.865 mm,−1], respectively. It was found that the FWHMs of the light distribution of the two points are 5.69 mm and 5.73 mm, respectively, and the overlapping position of the two points are at their FWHMs. This suggests that the resolution of the image is indeed close to 5.70 mm.

Some recent research has indicated that fluorophores emitting light at longer wavelength at first or second NIR window can achieve a better spatial resolution [31], and so the differences between the research conducted here and these papers are worth discussing. Although the published data refers to deep tissue imaging, the results demonstrated are still in light-scattering regimes where there is a significant proportion of weakly scattered light present, where change in the scattering coefficient has a significant effect and imaging resolution is still determined by the laws of geometrical optics. For example, the authors of [32] indicate that to obtain a sharp image the focal plane of imaging using NIR fluorophores needs to be a few millimetres under the surface of the sample owing to the deeper penetration depth of the NIR photons. The cases simulated in this paper are in the diffuse imaging regime where multiply scattered light dominates and geometrical optics is no longer applicable. This would be the case for imaging many organs of small animals. Further improvements to spatial resolution could be obtained by isolating weakly scattering photons from the majority of the multiply scattered light by temporal gating [33]. However, this considerable increases the complexity of the detectors used.

The signal-to-noise ratio (SNR) of a practical system will depend on the surface radiance and the choice of photodetector (e.g., photomultiplier tube, CCD or CMOS camera). Given the wide range of available commercial detectors, surface radiance is plotted to provide general results that can be utilised for specific detectors. Appendix B describes how surface radiance can be used to calculate SNR depending on the detector.

A small animal fluorescence imaging system which can provide scanning illumination can be built based on the simulation. Two galvanometer mirrors can be used to deflect a laser beam to provide the scanning capability. The fluorescent emission will be captured by a photon detector (e.g., a PMT) mounted in a fixed position. Note that, although it is time-consuming to obtain a scanned image from the simulation (~6 h depending on the computing capability), the speed to obtain one image from the setup will be fast. The scan rate of a galvanometer mirror is approximately 1–100 kHz [34]. To obtain an image with an illumination area of [−22 22] with a scan step of 1 mm, as simulated in this paper, the acquisition time can be as short as 2 s for a scan rate of 1 kHz. The acquisition time may be extended slightly if averaging is required to increase SNR of the detector; however, it is still practical for imaging of anaesthetised small animals.

## 5. Conclusions

In summary, this study numerically investigated the influence of the optical properties and imaging configurations on the surface radiance and spatial resolution of fluorescence imaging in tissue. For the full-field illumination system, a lower $\mu_{a}$ results in a higher surface radiance but the spatial resolution is worse. Many previous studies focused on imaging at NIR wavelength because of its deep penetration depth. This results in a stronger signal; however, there is a trade-off between the detected light and the spatial resolution. The spatial resolution of the scanning system is independent of $\mu_{a}$ at the emission wavelength. In addition, the simulation indicates that the spatial resolution of the scanning system is better than full-field illumination system. The spatial resolution of the scanning system is influenced only by the illumination light (i.e., distance between the targets and the excitation source, excitation wavelength). Both the detection mode and the fluorescence emission wavelength have no effect on its spatial resolution.

## Figures and Tables

**Table 1:** Parameters of quantum dots (QDs).

Symbol	Meaning	Value
$\xi^{ex}$	QDs molar extinction coefficient	$2\times 10^{4}$$M^{-1}\;{\mathrm{cm}}^{-1}$ [20]
Φ	QDs quantum yield	0.6 [21]
$N_{0}$	QDs concentration	$1\times 10^{20}$ $m^{-3}$

## Appendix A. Point Spread Function

Example PSFs for the scanning system are shown in Figure A1 ($\lambda_{ex}=600\;\mathrm{nm}$, $\lambda_{em}=1000\;\mathrm{nm}$ and location of the point is [0 0 −1] mm) and Figure A2 ($\lambda_{ex}=600\;\mathrm{nm}$, $\lambda_{em}=700\;\mathrm{nm}$ and location of the point is [0 0 −1] mm).

The estimated resolutions for a range of excitation wavelengths with fixed emission wavelength are summarised in Table A1. Location of the point target is [0 0 −1] mm.

**Table A1:** Resolution for a range of excitation wavelengths for scanning system. $\lambda_{em}=1000\;\mathrm{nm}$.

$\lambda_{\mathrm{ex}}$	$\mu_{s^{\prime }}$	$\mu_{a}$	FWHM Trans	FWHM Reflec
600 nm	1.49 mm	0.1504 mm	5.76 mm	5.76 mm
700 nm	1.23 mm	0.0162 mm	8.84 mm	8.84 mm
800 nm	1.03 mm	0.0220 mm	8.75 mm	8.75 mm
900 nm	0.89 mm	0.0304 mm	8.60 mm	8.60 mm

The spatial distribution of the fluorescence emission light of the first target, the second target, the superposition of the two images of the single targets, and the two targets are shown in Figure A3. The coordinates of the first target and the second target are [0,−2.865 mm,−1] and [0,2.865 mm,−1], respectively. $\lambda_{ex}=600\;\mathrm{nm}$, $\lambda_{em}=700\;\mathrm{nm}$. The FWHMs of the light distribution of the two points are 5.69 mm and 5.73 mm, respectively, and the overlapping position of the two points are at their FWHMs.

## Appendix B. Calculation of SNR

The SNR of the full-illumination system which uses a CCD camera as the detector can be calculated by [35]

(A1) $$SNR\equiv \frac{S_{e}}{N}=\frac{S_{e}}{\sqrt{S_{e}+R_{N}^{2}+D_{c}}}$$

where $S_{e}$ is the signal (electrons/pixel), $R_{N}$ is the read noise and $D_{c}$ is the noise associated with dark charge. $S_{e}$ can be calculated as

(A2) $$S_{e}=LdAd\Omega Q_{E}\tau$$

where *L* is the surface radiance, dA is the pixel area element at the object plane, dΩ is the collection solid angle, $Q_{E}$ and τ are the quantum efficiency and the integration time of the CCD camera respectively.

The scanning system uses an integrated detector which usually is a PMT. Therefore, the SNR of the scanning system can be calculated with the formula for SNR of the cathode current of a PMT [36]:

(A3) $$SNR=\frac{i_{s}}{\sqrt{2q(i_{d}+i_{s})B}}$$

where $i_{s}=2\pi IqQ_{EPMT}$ is the photocathode signal current, *I* is the light intensity at the detector, *q* is elementary charge, $Q_{EPMT}$ is the quantum efficiency of the PMT, $i_{d}$ is the dark current of the PMT and *B* is the equivalent noise bandwidth of the detection circuitry.

Typical parameters for SNR calculations for the IVIS system [35] and PMT [36] are shown in Table A2.

**Table A2:** Parameters used in signal-to-noise ratio (SNR) calculation.

Symbol	Meaning	Value
$R_{N}$	read noise of the CCD	5 electrons
$D_{c}$	dark current of the CCD	0.01τ electrons/pixel
dA	pixel area element of the CCD at the object plane	0.00021 cm^2^
dΩ	collection solid angle of the CCD	π/4 steradian
$Q_{E}$	quantum efficiency of the CCD	0.85
τ	integration time of the CCD	60 s
$Q_{EPMT}$	quantum efficiency of the PMT	0.3
$i_{d}$	dark current of the PMT	$10^{-15}$ A
*B*	bandwidth of the PMT	3 MHz
